# “Returning to Sport Is Not Just About the Knee”: Physiotherapists’ Experiences of the Management of Anterior Cruciate Ligament Injury: A Qualitative Study

**DOI:** 10.3390/jcm14207301

**Published:** 2025-10-16

**Authors:** Sultan A. Alanazi, Samia A. Alamrani, Sarah S. Bajuaifer, Layan Alhammad, Nouf Alotaibi, Naif Z. Alrashdi, Msaad Alzhrani, Ahmad D. Alanazi, Ahmed M. Almansour, Abdulmajeed Alfayyadh, Aqeel M. Alenazi

**Affiliations:** 1 Department of Physical Therapy and Health Rehabilitation, College of Applied Medical Sciences, Majmaah University, Al-Majmaah 11952, Saudi Arabia; LayannDPT@gmail.com (L.A.); Noufenadalotaibi@gmail.com (N.A.); n.alrashedy@mu.edu.sa (N.Z.A.); m.alzhrani@mu.edu.sa (M.A.); aalanazi@mu.edu.sa (A.D.A.); ah.almansour@mu.edu.sa (A.M.A.); 2 Department of Health Rehabilitation Sciences, Faculty of Applied Medical Sciences, University of Tabuk, Tabuk 71491, Saudi Arabia; salamrani@ut.edu.sa; 3 Department of Rehabilitation Sciences, College of Health and Rehabilitation Sciences, Princess Nourah Bint Abdulrahman University, P.O. Box 84428, Riyadh 11671, Saudi Arabia; ssbajuaifer@pnu.edu.sa; 4 Health and Basic Sciences Research Center, Majmaah University, Al-Majmaah 11952, Saudi Arabia; 5 Department of Physical Therapy and Health Rehabilitation, College of Applied Medical Sciences, Jouf University, Sakaka 72346, Saudi Arabia; abalfayyadh@ju.edu.sa; 6 Department of Health and Rehabilitation Sciences, College of Applied Medical Sciences, Prince Sattam Bin Abdulaziz University, Al-Kharj 11942, Saudi Arabia; aqeel.alanazi@psau.edu.sa

**Keywords:** rehabilitation, psychological readiness, return to sport, exercise therapy, fear of re-injury, athletic injury, Saudi Arabia

## Abstract

**Objectives:** To explore sport physiotherapists’ perspectives and experiences on the management of anterior cruciate ligament (ACL) injuries in Saudi Arabia and to understand the key challenges that influence rehabilitation practice. **Methods:** A qualitative semi-structured interviews were conducted with sport physiotherapists recruited from different regions and clinical settings (Public, private and sport clubs) in Saudi Arabia. Eligibility criteria included ≥2 years’ post-qualification experience in ACL injuries management. Interviews were audio-recorded, transcribed verbatim, and analyzed using reflexive thematic analysis. A total of twenty-six sport physiotherapists (18 males; mean age 31 years, range 26–39) participated, including 13 working primarily as clinicians and 13 with combined clinical and research roles. **Results:** Five themes were identified: (1) disruptions of ACL injury in daily life and sport; (2) managing fear of re-injury and uncertainty in surgical vs. conservative care; (3) guiding rehabilitation through challenges of workload, progression, adherence and supportive tools; (4) variability in protocols, assessment tools, and available resources; and (5) returning to sport is more than just passing a test. These themes demonstrate that ACL injuries rehabilitation in Saudi Arabia is shaped by physical and psychological readiness, cultural expectations, and disparities in resources. **Conclusions:** Physiotherapists described ACL rehabilitation as a long-term process that needs an integration of physical, psychological, and contextual factors. This study’s findings highlight the need for resource-sensitive, standardized guidelines and the inclusion of psychological readiness assessments within rehabilitation processes. This study provides context-specific evidence that can be used to inform the development of culturally responsive, evidence-based approaches to strengthen ACL rehabilitation in Saudi Arabia.

## 1. Introduction

Anterior cruciate ligament (ACL) injuries are among the most prevalent and debilitating musculoskeletal conditions in sport [1]. Globally, more than two million ACL injuries occur annually, with incidence rate among athletes exceeding 350 per 100,000 person-years, which is higher compared to the general population [2,3,4,5]. Athletes engaged in pivoting and high-intensity sports are particularly vulnerable to ACL injuries, which may result in long-term mechanical/health consequences such as knee instability, early knee osteoarthritis onset, reduced sport performance, and/or psychological distress [6,7,8]. Rehabilitation plays a critical role in ACL recovery; however, outcomes remain inconsistent, particularly regarding return to sport success rates and long-term knee health [9,10].

In Saudi Arabia, ACL injuries have become a growing sport injury concern, especially among soccer players and young athletes [11]. Prevalence rates of ACL injury in Saudi population have been reported as high as 26.2% [11,12,13,14]. Prior research suggest that ACL ruptures account for up to 20% of all sports-related knee injuries, with a steady increase in ACL reconstruction procedures has been observed in major hospitals over the last decade [14,15,16,17]. While surgical management for ACL rupture is common (especially for athletes), post-operative rehabilitation outcomes remain variable [18,19]. This variability could be due to several factors such as differences in clinician expertise, lack of access to specialized rehabilitation services, discontinuity of care, and/or disparities between public and private healthcare sectors in terms of the implementation of clinical practice guidelines [18,19,20,21]. In addition to the previous variability, the Saudi healthcare context is also influenced by cultural factors (e.g., family and community expectation) as well as systemic factors such as healthcare structure and resource availability [22].

Globally, there is growing recognition that successful ACL rehabilitation must address not only physical restoration but also psychological and contextual factors [9,23]. However, clinical implementation often deviates from evidence-based standards [18,23]. For example, a 2023 qualitative study of 19 physiotherapists in the United Kingdom highlighted significant variability in ACL rehabilitation delivery, describing access to care as a “location lottery” shaped by disparities in training, resources, and multidisciplinary coordination [24]. Similarly, Piussi et al. reported that sports physiotherapists’ often feel underprepared to address the psychological burden of ACL injury, despite acknowledging its importance in rehabilitation success [25]. These previous findings highlight the need for more context-specific research in healthcare systems such as Saudi Arabia’s where structural and cultural challenges remain underexplored.

Physiotherapists play a critical role in ACL rehabilitation, but little is known about their perceptions and experiences of clinical practice within the Saudi healthcare system. ACL injuries impose substantial societal and economic costs, often exceeding USD 10,000 per case when considering surgery, rehabilitation, and work absenteeism [26,27]. Global and regional disparities in access to specialized rehabilitation and inconsistent adherence to evidence-based practice further complicate recovery, particularly outside main urban centers [28,29]. In Saudi Arabia, limited access to specialized rehabilitation services further compounds these challenges, leading to delayed return to sport or work. Understanding these perspectives is vital given the broader public health, economic, and service implications of ACL injuries such as the costs of delayed return to sport or work, unequal access to specialized rehabilitation, and the psychological consequences of prolonged inactivity. Therefore, this qualitative study aimed to explore how sport physiotherapists in Saudi Arabia perceive and manage ACL injuries and to identify the contextual and systemic factors influencing rehabilitation practice. We proposed that psychological and structural factors shape physiotherapists’ application of return to sport criteria, which may contribute to variability in clinical decision-making.

## 2. Methods

### 2.1. Study Design

A descriptive qualitative design was employed to explore the perceptions and experiences of sport physiotherapists involved in the ACL rehabilitation in Saudi Arabia. The study was approved by the Institutional Review Board at Majmaah University (Approval #: MUREC-Dec.25/COM-2024/67). All participants provided informed consent prior to interviews. This study was reported in accordance with the COnsolidation criteria for REporting Qualitative research (COREQ) (Supplementary File) [30].

### 2.2. Participants and Recruitment

Eligible participants were licensed sport physiotherapists currently practicing in Saudi Arabia with at least 2 years of experience managing individuals with ACL injuries. Inclusion criteria were: (1) age ≥ 18 years, (2) qualified as a sport physiotherapist, (3) minimum of two years’ post-qualification clinical experience in ACL rehabilitation, and (4) proficiency in English and/or Arabic.

To capture diverse insightful perspectives from participants, recruitment sought variations across sex, years of experience, clinical settings (i.e., public, private, and sport club), and geographical regions [31]. Recruitment took place from January to July of 2025 using purposeful and snowball sampling techniques. Study announcements were distributed through social media platforms (e.g., X and LinkedIn) where a brief description of the study and a short eligibility survey were provided. Eligible respondents who completed the eligibility survey and met the selection criteria were contacted via email or phone call, according to their preferred method indicated in the short survey and invited to participate in semi-structured interviews. Additionally, interviewed physiotherapists were asked to recommend colleagues with ACL injuries expertise.

### 2.3. Sample

Twenty-six physiotherapists participated in this study. Their characteristics are presented below in Table 1.

### 2.4. Data Collection

Semi-structed interviews were conducted via Zoom call (Version 6.5.9, San Jose, CA, USA: Zoom Video Communications Inc.) using an interview guide (Supplementary File). The interview guide, eligibility notice, and invitation script are provided in a Supplementary File. The interview guide was developed collaboratively by the authorship team including senior physiotherapy researchers with experience in ACL injuries management, have Master of Musculoskeletal rehabilitation and PhDs in physiotherapy and have previously published qualitative research [32,33,34,35]. The interview guide facilitated open discussion regarding sport physiotherapists’ experiences with ACL injury rehabilitation. The guide included broad, exploratory prompts specifically designed to elicit participants’ perspectives on patient needs, rehabilitation practices, contextual challenges, and return to sport considerations. The guide was piloted with two expert sport physiotherapists to assess clarity, relevance, and flow, and minor revisions were made accordingly. Demographic data were collected on age, sex, current employment role (e.g., researcher, clinician), years of clinical experience, practice sector (governmental or private), highest qualification, and the number of surgical and non-surgical ACL cases seen in the past month.

At the start of each interview, the interviewers explained study procedures, addressed any participants queries and clarified confidentiality, the voluntary nature of participation, and the right to withdraw at any time. To reduce social desirability and confirmation bias, participants were assured of confidentiality, informed that there were no right or wrong answers, and interviews used neutral, non-judgmental prompts and non-leading open-ended questions. The Interviews were conducted by two final year female Doctor of Physical Therapy students (LA and NA) who had received extensive qualitative research training from senior qualitative researchers (First, second and third authors) and were supervised by the first author (SAA). Prior to this study, the interviewers had no prior relationship with participants, and no field notes were taken during interviews. Interviews were audio-recorded and lasted between 16 and 41 min and only the interviewer and participant attended (except the first 2 interviews which were observed by the first author to ensure clarity and appropriateness of data collection process). At the end of each interview, participants were asked to share any additional thoughts or ideas that have not been covered during the interview. All interviews were conducted once, and no repeated interviews were required.

### 2.5. Data Analysis

Interviews were conducted in Arabic, audio-recorded, professionally transcribed verbatim and translated into English by two senior and bilingual authors (First and third authors), who have Master of Musculoskeletal Physical Therapy and PhDs in physiotherapy and have previously conducted qualitative research. Transcriptions were independently cross-checked for accuracy and appropriateness, after which the authors met to compare and resolve any discrepancies. The interview data were analyzed using the six-stage model reflexive thematic analysis described by Braun and Clarke [36,37]. This model involved familiarizing, finding, analyzing, categorizing, comparing, and describing codes to generate themes that capture the phenomena under investigation and addressed the research aim [36,38].

Coding was conducted line-by-line to identify potential themes in accordance with the aim of this study. The third author (SSB) independently coded the first five interviews and developed a coding workbook using Microsoft Excel, which was independently reviewed by the first author (SAA) to ensure accuracy and to provide additional modifications. The codes were then checked and verified by the second author (SAA) who has experience in qualitative research, as this step is to ensure clarity and appropriateness of the codes generated. The third author (SSB) then coded the full dataset and produced an Excel spreadsheet of codes and emerging themes. The first author (SAA) independently reviewed and refined codes and themes and discrepancies were resolved through discussion. Data saturation was deemed to have been achieved when additional interview data produced little to no change in the emerging codes from the analysis [36]. Investigator triangulation was employed to enhance the rigor of the analysis and ensure a complete and in-depth interpretation of the data.

All research team reviewed and agreed upon the final list of themes identified. For respondent validation [39], preliminary themes were shared with participants for feedback and verification. Feedback received confirmed that the themes accurately represented participants’ perspectives, supporting the credibility and trustworthiness of the findings. All de-identified data were securely stored on a password-protected server with restricted access to the research team only.

### 2.6. Researcher Reflexivity

The authorship team comprised physiotherapists and academics with expertise in musculoskeletal rehabilitation, qualitative methods, and sports physiotherapy. Their shared professional background provided valuable insight into ACL injury rehabilitation but also carried potential preconceptions about clinical practice in Saudi settings. To address this, reflexive discussions were maintained throughout data collection and analysis to identify and challenge assumptions. Regular peer debriefing enhanced interpretive rigor and transparency. This reflexive stance acknowledges researcher influence as integral to qualitative inquiry and contributes to the study’s credibility and trustworthiness [40].

## 3. Results

### 3.1. Identified Themes That Describe the Sport Physiotherapists Perception Managing Individuals with ACL Injuries

Five overarching themes were identified from the analysis of interviews with sport physiotherapists managing individuals with ACL injuries: (1) disruptions of ACL injury in daily life and sport; (2) managing fear of re-injury and uncertainty in surgical vs. conservative care; (3) guiding rehabilitation through challenges of workload, progression, adherence and supportive tools; (4) variability in protocols, assessment tools, and available resources; and (5) returning to sport is more than just passing a test. Each theme is described in detail in the sections below with subthemes (*italicized*) for clarity.

#### 3.1.1. Theme 1: Disruptions of ACL Injury in Daily Life and Sport

Sport physiotherapists consistently described ACL injuries as profoundly disruptive, affecting patients’ daily routines, sporting identities, and social participation. Several participants observed that their patients struggled with basic activities such as kneeling, sitting cross-legged, or praying, which often limited engagement in both daily and cultural practices. The disruption was particularly evident among athletes, as one participant noted: *“An ACL injury affects the quality of life… especially for those involved in sports”* (P1).

Many sport physiotherapists highlighted the psychological consequences of ACL injuries, describing patients’ fear, frustration, and uncertainty about recovery, often asking questions whether they would return to sport or trust their knee again. For elite athletes, some participants reported that prolonged time away from sport had major negative impacts on identity and livelihood: *“being off the field for 6 to 9 months causes a huge psychological impact on athletes”* (P23).

Social consequences were felt by many sport physiotherapists, who observed that their patients often avoided family or community gatherings to escape questions such as *“Will you stop playing football?”* (P25). Being away from the team environment was perceived as particularly discouraging for athletes. While financial burdens were generally minimal in public hospitals (government-funded care), many participants acknowledged broader economic impacts on patients and the healthcare system. Collectively, these disruptions covered physical, psychological, social, and economic domains (*Physical disruption, psychological effect, social consequences, Economic impact;* Table 2).

#### 3.1.2. Theme 2: Managing Fear of Re-Injury and Uncertainty in Surgical vs. Conservative Care

Participants frequently expressed that fear of re-injury often dominated clinical reasoning and strongly influenced patients’ treatment decisions. Fear was described as a psychological barrier shaping decision-making—particularly when selecting between surgical and conservative options. Several sport physiotherapists noted that their patients often carried lasting memories of the initial trauma, describing *“hearing a pop in the knee”* (P11) or saying that *“their knee feels disconnected”* (P11). The persistent fear of re-injury often limited patients’ engagement in rehabilitation and, in some cases, led them to pursue surgical reconstruction despite available conservative options. One physiotherapist explained that *“the main fear is not being able to return to their sport… some assume they’ll have to quit completely”* (P18). Another participant added that *“fear of re-injury is the most common psychological barrier… patients are often afraid of going through the same injury again”* (P10), emphasizing that confidence in the knee extends beyond structural healing and remains central to treatment decision-making.

Uncertainty about long-term outcomes was also reported by several participants. They explained that their patients frequently asked whether they could ever fully recover, whether osteoarthritis would inevitably develop, or whether surgery could guarantee a successful return to sport. Sport physiotherapists noticed that many patients carried unrealistic expectations, particularly regarding surgical outcomes: *“some of the patients want surgery because they believe it will solve everything, but the reality is much more complicated”* (P7).

These anxieties were not limited to elite athletes; recreational and physically active individuals expressed similar concerns about long-term limitations. Several sport physiotherapists described their role as an ongoing negotiation—providing reassurance while correcting misconceptions about both surgical and non-surgical pathways. Ultimately, treatment decisions were often shaped as much by fear, uncertainty, and psychological stress as by medical advice (*fear of re-injury, uncertainty about outcomes, psychological stress;* Table 3).

#### 3.1.3. Theme 3: Guiding Rehabilitation Through Challenges of Workload, Progression, Adherence, and Supportive Tools

Several sport physiotherapists consistently emphasized that ACL rehabilitation is long, exercise-driven, and requires sustained commitment from patients. Exercise was described as the cornerstone of management: *“from my side, the most important intervention is exercise… especially early on”* (P18). Early rehabilitation typically involved Isometric and endurance strengthening, progressing to concentric, eccentric, and plyometric drills. Adjunctive modalities such as electrotherapy, hydrotherapy, blood-flow restriction, or shockwave were occasionally applied, but always secondary to progressive loading.

Managing progression through phases was described as uneven and clinically challenging. Initial priorities included reducing swelling, restoring quadriceps activation, and regaining range of motion, particularly knee extension. Many participants repeatedly emphasized extension as decisive for outcomes: *“the hardest issue is achieving full extension. Flexion comes back, but extension must be addressed early”* (P9). Failure to restore extension promptly was associated with adhesions, fibrosis, or even the need for secondary surgery.

Weight-bearing strategies varied according to injury characteristics and surgical details. Some participants encouraged early loading when clinically appropriate: *“if the ACL tear is isolated, early weight-bearing is encouraged… gradual progression is essential in all cases”* (P9). In contrast, others delayed weight-bearing following meniscal repair or when graft-related restrictions were present.

Rehabilitation trajectories differed between surgical and non-surgical patients. Some sport physiotherapists observed that non-surgical cases often progressed more quickly and regain muscle strength sooner, while surgical patients followed longer timelines and were often more cautious: *“surgical patients usually need six months; non-surgical may recover in one or two”* (P4). Despite this, surgery was frequently perceived to provide greater long-term stability in the knee joint.

Adherence throughout rehabilitation phases was seen as the most persistent challenge, caused by fear of movement, competing responsibilities, or limited understanding of goals: *“patient adherence to the treatment plan is my top concern… lack of commitment can significantly delay recovery”* (P14). Miscommunication between surgeons and physiotherapists regarding rehabilitation phases and timeline was reported as a limiting factor that can further complicate adherence.

Supportive tools, particularly knee braces, were used selectively. Few participants prescribed them in the early post-surgical phase to provide confidence and protect the joint: *“braces are helpful early on for safety and maintaining extension”* (P6). Others discouraged routine use, cautioning against dependency and stiffness: *“personally, I’m not a fan of knee braces unless there’s a strong reason…. they can limit recovery if overused”* (P2).

Post-surgical complications, including swelling, arthrofibrosis, cyclops lesions, and infections, were reported as significant barriers to progress, reinforcing the need for early, structured, and closely monitored rehabilitation.

Overall, sport physiotherapists described ACL rehabilitation as a negotiated, exercise-driven process shaped by progression management, adherence challenges, use of supportive tools, and risks of complications. These findings clustered around the following subthemes: (*common interventions, progression of therapy, weight-bearing strategies, surgical versus non-surgical recovery, post-surgical complications, adherence challenges, and regaining knee extension;* Table 4).

#### 3.1.4. Theme 4: Variation in Protocols, Assessment Tools and Resources

Many sport physiotherapists consistently reported wide variation in how ACL rehabilitation protocol was delivered, both within and across clinical settings *“protocols vary between hospitals and private clinics. Generally, implementation in private clinics is better, especially in terms of time and flexibility.”* (P19). While some reported that they adhere to internationally recognized guidelines, others relied mainly on personal experience, leading to inconsistent management practices. One participant reflected, “*protocols vary from therapist to therapist. I personally follow the Melbourne Protocol…which I see as reliable for gradual rehabilitation”* (P9). This lack of a unified approach was seen as a major barrier to ensuring consistent quality of care.

It is reported that there was discrepancy in ACL assessment practices. Although a number of clinicians employed objective measures such as hop tests, strength assessment, or kinesiophobia scales many acknowledged that decisions were often based on clinical observation rather than standardized testing. Limited time and restricted access to specialized equipment, particularly in high-volume government hospitals, were reported as key challenges.

Many sport physiotherapists reported that the resource disparities have influenced rehabilitation practices. Several participants in private centers reported access to advanced equipment and technologies, whereas those in public hospitals frequently encountered shortages of even basic tools. These differences not only affected rehabilitation trajectories but also shaped clinicians’ perception of their ability to provide optimal care. These accounts highlight an ongoing challenge of inconsistency in ACL injury rehabilitation and underscore the need for standardized clinical guidelines. Overall, accounts revealed marked inconsistency in protocols, outcome measures, resource limitations, highlighting the need for standardized, context-sensitive clinical guidelines that account for variations across healthcare settings (*Protocol variability in practice, outcome measures, resource limitations;* Table 5).

#### 3.1.5. Theme 5: Returning to Sport Is More than Just Passing a Test

Return to sport was consistently viewed as a process that extends beyond passing a physical test, with fear of re-injury emerging as a key determinant of psychological readiness and confidence rather than a treatment barrier. While measures such as hop tests and strength assessments were regarded as essential benchmarks, many physiotherapists reported that psychological readiness often lagged behind physical recovery. As one participant explained, “*they may pass all the tests, but still tell you they don’t feel ready”* (P14). This mismatch between physical clearance and perceived readiness created ongoing challenges in clinical decision-making.

External pressures further complicated the process. Athletes were often urged to return prematurely by coaches, clubs, or families, even when they were not psychologically prepared. One participant noted, *“some athletes rush because they don’t want to lose their spot on the team”* (P15). These competing demands often placed sport physiotherapists in the difficult position of balancing immediate performance with long-term safety.

Many of our participants stressed that successful return to sport required more than biomechanical recovery. Confidence in the knee, trust in performance, and gradual reintegration into competition were seen as equally critical. As one therapist summarized, *“returning to sport is not just about the knee…it’s about the whole athlete being ready again”* (P15). Collectively, these accounts underscore that return to sport cannot be reduced to test results alone but must incorporate physical readiness, psychological preparedness, and the broader social and cultural context (*Physical readiness, psychological preparedness, external pressures, social and cultural context;* Table 6).

## 4. Discussion

This is the first qualitative study that explored the perspectives of sports physiotherapists on the management of ACL injuries working in Saudi Arabia. This study findings revealed that sport physiotherapists view ACL rehabilitation as a complex, multidimensional process that extends beyond physical recovery and incorporates psychological, social and contextual considerations. Participants emphasized that the disruption ACL injuries caused in daily and sporting activities, the impact of fear of re-injury and uncertainty in treatment decisions, the difficulties of sustaining progression and adherence within long rehabilitation pathways, and the variability in protocols, assessment tools, and resources across settings. Sport physiotherapists also stressed that returning to sport requires more than passing physical tests, as psychological preparedness and cultural context are equally decisive. The five main themes highlighted the clinical and contextual realities of ACL rehabilitation in Saudi Arabia and extended recent qualitative research from Europe and Australia [24,25,41,42]. These themes offer insights into the complex realities of clinical practice and point out key areas for improvement in care delivery and policy development.

### 4.1. Theme 1: Disruptions of ACL Injury in Daily Life and Sport

Based on participants’ responses, ACL injuries were described as extending beyond the clinic environment, disrupting daily life and requiring ongoing adaptation. Sport physiotherapists observed that their patients with ACL injuries often struggled with restrictions in basic activities, altered social roles, and psychological adjustment and in some cases financial stability. This is similar to the recent work describing ACL reconstruction as a “life-shaping event” rather than a single surgical episode [41,42]. Such disruption is not only peripheral but central, shaping how athletes engage in rehabilitation from the outset. The psychological burden of ACL injury was particularly notable among athletes, where long rehabilitation timelines challenged identity and career prospects [43,44]. These disruptions highlight the importance of adopting a biopsychosocial approach in ACL rehabilitation. Recognizing that recovery is not only biomechanical but also highly individualized and contextual [45,46,47].

### 4.2. Theme 2: Managing Fear of Re-Injury and Uncertainty in Surgical vs. Conservative Care

A major finding of this study was that the fear of re-injury emerged as a central factor influencing decision-making, often persisted despite favorable physical progress, consistent with previous studies [48,49]. Our participants noted that readiness to return to sport was not always aligned with test results and was frequently shaped by uncertainty and external pressures from coaches or families. These observations mirror evidence that psychological readiness is strongly associated with return to sport status, with those who return typically reporting higher ACL-return to sport scores than non-returners, and that paths of readiness may stagnate for long periods without targeted input [50,51]. Importantly, prior qualitative research has indicated how fear is constructed and maintained, underscoring why it can dominate decisions even when physical criteria are met [50]. A 2025 study suggested that the timing of measuring psychological readiness is important: administering the ACL–return-to-sport questionnaire after completing physical performance tests, may provide more realistic appraisal of self-confidence than pre-test questionnaires [52]. Therefore, sport physiotherapists play a dual role, not just as providers of physical rehabilitation, but also as emotional supporters and educators. They are responsible for addressing misunderstandings, providing reassurance, and guiding patients to make informed, evidence-based decisions rather than fear [53,54].

### 4.3. Theme 3: Guiding Rehabilitation Through Challenges of Workload, Progression, Adherence, and Supportive Tools

Study participants reflected on the tension between criterion-based rehabilitation and the practical realities of adherence. Fluctuating motivation, socioeconomic barriers, and competing responsibilities often disrupted structured progression, despite physiotherapist follow contemporary guidelines that emphasize criteria-based loading, progressive strength training, and staged decision points across the continuum [23]. This clinical picture is consistent with broader evidence on the adherence barriers in ACL rehabilitation, including logistical constraints, low self-efficacy, and environmental pressures [55,56]. Highlighting the need to embed behavior-change strategies and supportive tools into routine care [55,57].

### 4.4. Theme 4: Variation in Protocols, Assessment Tools and Resources 

A key systemic challenge identified in this study was the variation in assessment practices and resource availability, which added further complexity in ACL rehabilitation. Sport physiotherapists described inconsistent use of hop tests, strength measures, and movement-quality ratings, reflecting a broader lack of alignment on benchmarks and clearance criteria. This inconsistency has also been noted internationally. This aligns with international evidence showing substantial heterogeneity in return-to-sport testing and decision-making, even among expert clinicians [24,58]. A recent Delphi consensus study further highlights the need for standardized, multimodal assessment protocols to guide safe progression [58]. In addition, mounting evidence cautions that hop-test limb-symmetry indices can overestimate functional recovery relative to strength testing and that adding movement-quality ratings improves decision signal beyond distance-based metrics [59,60]. Taken together, these findings support the move toward multimodal, serial testing rather than one-off pass/fail decisions [61].

### 4.5. Theme 5: Returning to Sport Is More than Just Passing a Test

Participants consistently reported that returning to sport is a complex, staged process that goes beyond simply passing physical performance tests, with psychological readiness often lags behind physical recovery. Sport physiotherapists emphasized how contextual factors including athlete identity, competitive demands, and coach or family influence, shape return to sport decision as much as physical criteria. These observations align with a very recent review and a qualitative study similarly suggesting that objective test batteries are necessary but insufficient, and that readiness must be interpreted alongside psychological, social and contextual information across staged decision-making [41,61]. Within the Saudi context, where competitive sport participation is rapidly expanding and social expectations can exert strong influence, these contextual factors may be particularly salient in shaping return to sport trajectories.

### 4.6. Strengths and Limitations Pertain to the Current Study 

This qualitative study followed the COREQ reporting guidelines [30], applied investigator triangulation and respondent validation, thereby increasing the trustworthiness of the study findings. The inclusion of sport physiotherapists from diverse clinical practice settings across both public and private healthcare settings in Saudi Arabia, provided insightful information about how ACL rehabilitation is adapted, strengthening the transferability of results.

However, several limitations should be considered. The purposive and snowball sampling approach may have introduced selection bias, participants with particular interest overrepresented, potentially limiting the diversity of perspectives. Each participant was interviewed only once, which may have limited the depth of inquiry. Field notes were not collected during interviews, potentially reducing contextual richness and may limit reflexivity regarding non-verbal cues. Although interviews were conducted by final-year DPT students who received training and regular supervision from senior qualitative researchers, their limited level of experience may have influenced the depth of probing during interviews. Additionally, as interviews were conducted primarily in Arabic, subtle nuances may have been lost during translation despite bilingual verification. Further, this study is based on data from Saudi sport physiotherapist only and does not include the views of athletes or coaches. Nonetheless, findings of this study provide important insights into sport physiotherapists’ experiences and perceptions that can support and guide the development of culturally tailored, patient-centered strategies for ACL injury management in Saudi Arabia. Future research should incorporate perspectives of athletes with ACL injuries and coaches and evaluate implementation strategies across healthcare sectors.

### 4.7. Implication for Practice

Sport physiotherapists experience and perceptions voiced in this study highlight several clinical implications that could strengthen ACL rehabilitation and return-to-sport outcomes. Incorporating a standardized measure of psychological readiness, such as the Anterior Cruciate Ligament–Return to Sport after Injury (ACL-RSI) scale, at planned stages of recovery may help identify athletes at risk of premature return [62]. Adopting criteria-based progression checklists that include strength, hop performance, and sport-specific tasks, rather than relying solely on time-based milestones, may support safer and more individualized rehabilitation. Clinicians who use criteria-based checklists tend to have better outcomes and lower re-injury rates than those relying mainly on arbitrary time milestones [21,62,63]. Ongoing professional development and interprofessional training are also needed to promote consistent, evidence-based practice across different healthcare settings (public and private) [62]. These steps would facilitate the alignment of clinical care with national priorities to improve rehabilitation standards and optimize outcomes for athletes in Saudi Arabia.

## 5. Conclusions

This study showed that sports physiotherapists in Saudi Arabia view ACL rehabilitation as a complex process shaped by lived disruption of sport and daily activities, fear of re-injury, adherence challenges, variability in practice, and the complexity of return to sport decisions. Sport physiotherapists emphasized that successful rehabilitation required more than applying standardized protocols, instead advocating for integration of physical, psychological, and contextual factors. The study findings highlight current practice challenges and emphasize the need for evidence-based guidance that can be adapted effectively within the Saudi healthcare system.

## Figures and Tables

**Table 1:** Participant characteristics (total n = 26).

Characteristic	Number (%)
Sex: Male	18 (69)
Age: Mean (range) years	31 (26–39)
Current employment role	
Clinician	13 (50)
Clinician and researcher	13 (50)
Years of clinical experience	
≤5	14 (54)
6–10	9 (35)
≥11	3 (11)
Sector of current clinical practice	
Governmental/semi-governmental	13 (50)
Private	13 (50)
Highest academic qualification	
Bachelor’s degree	16 (62)
Master’s degree	7 (27)
Doctor of Philosophy	3 (11)
Number of non-surgical ACL cases seen in last month	
1–5	21 (81)
6–10	4 (15)
>10	1 (4)
Number of post-surgical ACL cases seen in the last month	
1–5	15 (58)
6–10	7 (27)
>10	2 (8)
None	2 (8)

ACL = Anterior cruciate ligament. Unless otherwise stated, data are N (%).

**Table 2:** Subthemes and quotes for Theme 1: “Disruptions of ACL injury in daily life and sport”.

Subtheme	Illustrative Quote
Physical disruption	“ACL injury affects patients both physically and psychologically” (P9)
	“Regular activities like sitting cross-legged or prayer can become painful and difficult.” (P3)
	“I believe it has a significant impact… it affects their daily tasks, work duties, and responsibilities at home.” (P17)
	“ACL injuries impact athletes significantly…It can hinder their performance and return to competitive sports.” (P5)
Psychological effect	“The main fear is not being able to return to their sport… some athletes assume they’ll have to quit completely…” (P18)
	“ACL injury means being off the field for 6 to 9 months, which causes a huge psychological impact. Some athletes even go through abnormal levels of stress.” (P23)
Social consequences	“Some patients feel ashamed to be seen with crutches or feel weak. I remember one patient refused to attend a family gathering because he didn’t want to bring his crutch. In athletes, the impact is even greater.” (P23)
	“Socially, the injury can prevent the patient from fulfilling home or social responsibilities, leading to reduced interactions and isolation.” (P13)
Economic impact	“Economically, it has a burden on both the patient and the healthcare system.” (P11)

**Table 3:** Subthemes and quotes for Theme 2: “Managing fear of re-injury and uncertainty in surgical vs. conservative care”.

Subtheme	Illustrative Quote
Fear of re-injury	“Fear of re-injury is the biggest challenge.” (P10)
	“Fear of re-injury is the most common thing we hear, even more than pain.” (P14)
	“They always ask… ‘will I be the same again?’ they don’t trust the knee like before.” (P18)
Psychological stress	“Athletes lose months of their career, and with that comes anxiety, stress, and sometimes depression.” (P23)
	“The fear is not only physical… it’s emotional, because their career and identity are on the line.” (P19)
	“Patients worry a lot about re-injury, become cautious, reduce their movement, and often avoid sports or even daily activities like going up stairs or praying. It has a major psychological impact. (P16)
Uncertainty about outcomes	“Non-surgical cases recover quickly but often carry more fear of re-injury.” (P5)
	“Most patients are unsure what surgery or conservative treatment will bring; they’re worried about life after treatment.” (P22)
	“There’s always the question of what will happen after surgery… will they recover fully, or will they develop knee arthritis later?” (P7)
	“Pressure from family or peers to undergo surgery, especially when others had positive outcomes causes emotional conflict if the patient is unsure about the operation. (P5)

**Table 4:** Subthemes and quotes for Theme 3: “Guiding rehabilitation through challenges of workload, progression, adherence and supportive tools”.

Subtheme	Illustrative Quote
Common interventions (exercise as cornerstone)	“From my side, the most important intervention is exercise… especially early on.” (P18)
	“Isometric and endurance strengthening are the foundation before progressing to concentric or eccentric training.” (P19)
	“We follow standard protocols but may include modalities like hydrotherapy, electrotherapy, and muscle stimulation… but 95% is exercise.” (P20)
	“I mostly rely on exercise; in early stages, I add electrical stimulation or blood flow restriction if needed.” (P12)
Progression of therapy	“If extension is not restored in the first two months, the case becomes much harder.” (P11)
	“Each phase builds to the next… strength, then balance, then sport drills.” (P23)
	“Acute focuses on swelling and ROM; subacute is transition; chronic cases are more functional and goal driven.” (P7)
Weight-bearing strategies	“If the ACL tear is isolated, early weight-bearing is encouraged. Gradual progression is essential in all cases.” (P9)
	“Unless there’s a meniscus repair, early weight-bearing helps patients return to walking sooner.” (P11)
	“If meniscus is repaired, we restrict weight-bearing for 4–6 weeks.” (P3)
	“If symptoms are under control, I support early weight-bearing, especially for athletes.” (P16)
Surgical versus non-surgical recovery	“Surgical patients usually need six months; non-surgical may recover in one or two.” (P4)
	“Non-surgical patients often recover faster, with better muscle strength and fewer complications.” (P5)
	“Non-surgical cases progress quickly, but surgery provides more long-term stability.” (P22)
	“Patients without surgery regain strength faster, but surgical patients are more stable in the long term.” (P13)
Post-surgical complications	“The hardest issue is achieving full extension. Flexion comes back, but extension must be addressed early.” (P9)
	“Loss of extension even 5 degrees is serious. Delay full extension makes it much harder.” (P18)
	“Arthrofibrosis or cyclops lesions sometimes require a second surgery.” (P9)
	“Complications such as infections, I encountered a case of necrosis after surgery.” (P10)
Adherence challenges	“Non-compliance with rehab plans, fear of movement, and poor understanding of goals are common barriers.” (P25)
	“Some stop coming once pain reduces, which interrupts progression.” (P12)
	“The biggest barrier is adherence and lack of motivation… these delay recovery.” (P21)
Supportive tools	“Braces are helpful early on for safety and maintaining extension… they give patients confidence.” (P6)
	“Post-surgery, braces may help short-term but should not be used long-term.” (P21)
	“I support using them early on for confidence, but only for short-term.” (P24)

**Table 5:** Subthemes and quotes for Theme 4: “Variation in protocols, assessment tools and resources”.

Subtheme	Illustrative Quote
Protocol variability in practice	“No unified protocol… I adapt between Aspetar and Melbourne.” (P2)
	“Government hospitals stick to rigid monthly progressions; private settings allow flexibility.” (P19)
	“Protocols differ from therapist to therapist, and that leads to inconsistency.” (P2)
	“Some places follow strict guidelines; others go by experience… it’s very mixed.” (P16)
	“Some protocols are outdated, and patients get confused when advice changes between settings.” (P24)
Outcome measures	“We use hop tests, balance measures, goniometers, depending on stage of rehabilitation.” (P7)
	“For psychology, I use the Tampa Scale for Kinesiophobia… it matters as much as strength.” (P21)
	“We use hop tests, isokinetic measures, and sometimes VALD systems, but there’s no unified approach.” (20)
Resource limitations	“Some clinics don’t even have the basic tools… protocols stay on paper.” (P25)

**Table 6:** Subthemes and quotes for Theme 5: “Returning to sport is more than just passing a test”.

Subtheme	Illustrative Quote
Physical readiness	“Even after good physical recovery, athletes hesitate to return to pivoting sports.” (P15)
	“ACL injuries significantly impact athletes’ ability to return at the same level they once played.” (P19)
Psychological preparedness	“Fear of the knee failing is the top concern, even when physical tests look good.” (P9)
	Psychological readiness is a major factor (P25)
	“Many athletes are cleared physically, but mentally they’re not ready to trust their knee again.” (P23)
	“It’s our job as physiotherapists to reintroduce sport-specific activities like ball catching, sudden change direction, and jumping… to rebuild that trust.” (P11)
External pressures	“Coaches often push for quicker return to play… we have to advocate for safety.” (P12)
Social and cultural context	“Sometimes family pressure or social expectations influence the decision more than clinical tests.” (P13)
	“In some cases, cultural habits play a role… like avoiding certain movements even after clearance.” (P21)

## Data Availability

The datasets analyzed in the current study are available from the corresponding author on reasonable request.

## Supplementary Materials

The following supporting information can be downloaded at: https://www.mdpi.com/article/10.3390/jcm14207301/s1, The interview guide (English and Arabic).
