# Supplementary material for: “Returning to Sport Is Not Just About the Knee”: Physiotherapists’ Experiences of the Management of Anterior Cruciate Ligament Injury: A Qualitative Study
Source: J Clin Med. 2025 Oct 16;14(20):7301. doi: 10.3390/jcm14207301 (PMC12565288; doi:10.3390/jcm14207301)
Supplement: Supplementary file 1 [file jcm-14-07301-s001.zip › jcm-3914184-supplementary.pdf]

**1. Interview Guide / دليل المقابلة**

Introduction / المقدمة

Thank you for agreeing to take part in this interview and for giving us your time today. The interview will take approximately 15–20 minutes, during which I will ask you several questions about your experiences and perspectives regarding the rehabilitation of anterior cruciate ligament (ACL) injuries. Please note that the interview will be audio-recorded solely for data analysis purposes, and all information will remain confidential. There are no right or wrong answers; the main aim is to understand your experiences in managing patients with ACL injuries.

**Questions / الأسئلة**

**Overview and Clinical Presentation / نظرة عامة والحالة السريرية للمريض:**

- How do you think ACL injuries affect patients' lives?
- What are the most common concerns your patients with ACL injuries express?
- How does an ACL injury impact patients' physical activity and daily functioning?
- How does it affect their social, psychological, and economic aspects of life?
- What are the most common symptoms and signs among patients with ACL injury (without meniscal tear)?

**Rehabilitation / التأهيل**

- What are the most common therapeutic interventions your patients receive?
- What are the main differences between surgical and non-surgical patients?
- After surgery, what are the key concerns you focus on?
- What is the role of braces (Braces) in ACL management and why do you use them?

**Treatment Protocols / البروتوكولات العلاجية**

- What do you think about the ACL rehabilitation protocols currently applied in Saudi Arabia?
- Which protocol do you follow with your patients?
- How effective do you find it?
- What challenges have you faced applying it?
- What outcome measures do you use?
- How do you determine weight-bearing progression?

**Challenges and Barriers / التحديات والعوائق**

- What are the most common challenges ACL patients face?
- What challenges do they experience when returning to sport?
- When assessing fear avoidance, what tools do you use?
- How do you assess fear of re-injury?
- How do you manage patients with strong fear of re-injury?

Thank you very much for your participation. Before we conclude, is there anything else you'd like to add about your experience with ACL rehabilitation?

## Interview guide (Arabic)

**المقدمة:**

شكرًا لك  على موافقتك على عمل هذه المقابلة واعطائنا الوقت للقاء اليوم. مقابلتنا ستستغرق من 15 إلى 20 دقيقة وسأطرح عليك العديد من الأسئلة عن تجربتك وخبرتك في تأهيل اصابات الرباط الصليبي الامامي، نود أن نؤكد لك ان هذه المقابلة ستكون مسجلة صوتيا فقط، لغرض التحليل العميق للبيانات، ونؤكد لك كذالك ان هذه المعلومات ستكون سريه وخاصة فقط في اعضاء البحث المشاركين، ونؤكد لك انه لا يوجد اجابه صحيحه او خاطئة وان الغرض الاساسي هو معرفة تجربتكم وخبرتكم في تأهيل الاشخاص المصابين في بقطع الرباط الصليبي الامامي.

سيتم تسجيل هذه المقابلة، وقبل أن نبدأ، هل تفضل إجراء المقابلة باللغة الإنجليزية أم العربية؟

.إذا لم يكن لديك أي أسئلة، فلنبدأ الحديث

**الاسئلة:**

**:نظرة عامة والحالة السريرية للمريض**

1. تؤثر على حياة المريض؟ (ACL) كيف تعتقد أن إصابة الرباط الصليبي الأمامي
2. ما هي أكثر المخاوف شيوعًا لدى مرضاك المصابين بإصابة في الرباط الصليبي الأمامي؟

- كيف تؤثر إصابة الرباط الصليبي على النشاط الجسدي (الأنشطة اليومية) للمريض؟
- كيف تؤثر على الحياة الاجتماعية للمريض؟
- كيف تؤثر على الحالة النفسية للمريض؟
- كيف تؤثر اقتصاديًا؟
- ما هي جوانب الحياة المتأثرة بإصابة الرباط الصليبي؟

3. ما هي الأعراض والعلامات الأكثر شيوعًا التي يعاني منها المرضى المصابون بإصابة في الرباط الصليبي الأمامي (دون تمزق الغضروف الهلالي)؟

**التأهيل:**

4. ما هي التدخلات العلاجية الأكثر شيوعًا التي يتلقاها مرضاك المصابون بإصابة في الرباط الصليبي من خلال خبرتك؟
- من خلال خبرتك، ما الفرق الأكثر شيوعًا بين المرضى الذين خضعوا للجراحة والذين لم يخضعوا لها؟
- ما هي أكثر المخاوف التي تركز عليها عادةً بعد الجراحة؟

5. في علاج إصابات الرباط الصليبي ولماذا تستخدمها؟ (Braces) ما هو دور الدعامات

**العلاج:**

6. من خلال خبرتك، ما رأيك في بروتوكولات علاج إصابات الرباط الصليبي المطبقة في الممارسة السريرية في السعودية؟
- ما البروتوكولات التي تستخدمها مع مرضاك؟
- ما مدى فعالية البروتوكول الذي تستخدمه؟
- ما هي التحديات التي واجهتها عند استخدام هذا البروتوكول؟
- من خلال خبرتك، ما هي أدوات قياس النتائج التي تستخدمها مع مرضاك؟

7. ؟ هل يمكنك وصف كيف تحدد احتياطات تحميل (Weight Bearing) ما رأيك في دور تحميل الوزن الوزن وتقدّم المريض فيها ولماذا؟

**التحديات والعوائق**

8. ما هي التحديات الأكثر شيوعًا التي يواجهها مرضاك المصابون بإصابة في الرباط الصليبي؟
9. ما هي التحديات والمخاوف الأكثر شيوعًا التي يواجهها مرضاك عند العودة إلى ممارسة الرياضة؟

- (Fear avoidance) إذا كنت تقيّم خوف المرضى الذين خضعوا للجراحة من الحركة
- ما أدوات القياس التي تستخدمها؟
- كيف تقيّم الخوف من تكرار الإصابة؟
- كيف تتعامل مع المريض الذي يعاني من خوف كبير من تكرار الإصابة؟

**الخاتمه:**

- شكرًا لك على مشاركتكم، نقدر ونثمن وقتكم، هل يوجد هناك اي تجارب او خبرات عن تجاربكم مع تأهيل اصابات الرباط الصليبي الامامي، لم نتطرق لها من خلال هذه المقابله وتودون اضافاتها، نرحب بإي معلومات اضافيه تودون مشاركتها، - اخيرا هل يوجد اي وجهات نظر لديكم تفضلون اضافتها لهذه المقابلة، لم نتطرق اليها؟
-

مجددا، نشكر لكم عمل هذه المقابلة ونتمنى لكم يوما سعيداً

**2. Invitation Script (English)**

Dear Physiotherapist,
We are a research team from Majmaah University (Doctor of Physiotherapy Program) conducting a qualitative study that aims to explore physiotherapists' experiences and opinions on ACL rehabilitation.
To register your interest, please complete the 5-minute eligibility form via the link below.
Our team will contact you to schedule a 15–20-minute online interview.
Your data will be kept secure and confidential.
Thank you for your time and valuable contribution.

Invitation script (Arabic)

السلام عليكم ورحمة الله وبركاته،

نحن فريق بحثي تخصص دكتورعلاج طبيعي في جامعة المجمعة، نسعد بدعوتكم للمشاركة في هذه الدراسة النوعية والتي تهدف للاستفادة من ومعرفة خبرات وتجارب وآراء أخصائيين العلاج الطبيعي (المتخصصين في الاصابات الرياضيه) حول تأهيل إصابات الرباط الصليبي.

لتسجيل اهتمامكم ورغبتكم في المشاركة، الرجاء إكمال الاستبيان المؤهل للدراسه (5 دقائق لاكماله) في الرابط👇

سيتواصل معكم فريق البحث لتنسيق عمل المقابلة (15-20 دقيقة - عن بعد).

نؤكد لكم أن جميع البيانات التي تُجمع لهذا البحث سوف يتم الاحتفاظ بها في أمان وسوف تستخدم فقط للغرض الذي وافقت من أجله.

نثمن وقتكم وجهدكم ونشكركم على تعاونكم ومساهمتكم القيمة، ونسأل الله أن ينفع بهذه

# Physiotherapist’s experiences and perceptions about anterior cruciate ligament (ACL) injuries and rehabilitation in Saudi Arabia: A qualitative study

**Thank you for your interest in participating in our research.**

**Please read the following information carefully**

What is the purpose of the study?
This qualitative study aims to better understand physiotherapist's experiences, perceptions regarding ACL injuries and rehabilitation in Saudi Arabia.

This online survey is the first phase of this qualitative study. This online eligibility survey aims to assess the eligibility of potential participants. For those who are eligible we will invite them to complete the second phase, which is an online (via zoom) one-to-one interview for 15-20 minutes (depending on your response). The interview aims to understand sport physiotherapist’s experiences, and to gain their insights about managing ACL injuries with and/or without surgical interventions.
You are eligible to participate in this survey if you meet the following criteria:

- 18 years of age or older.
- qualified as a sport physiotherapist with a minimum of two years’ post-qualification clinical experience in ACL rehabilitation,
- Proficient in English and/or Arabic.

**Privacy & confidentiality?**
Your privacy will be maintained at all times, and you will not be identified, your data will be securely stored. If you have any questions about this research, please contact Dr Sultan Alanazi, PT, MSc, PhD (sa.alanazi@mu.edu.sa) or contact (DPT student Layan Alhammad, Majmaah University (layanndpt@gmail.com).

This study has obtained an ethical approval from Majmaah University (Approval #: MUREC-Dec.25/COM-2024/67).

**Please complete this form by answering the below questions.**

The form will take approximately 3-5 mins.

* Indicates required question

1. Are you a Saudi licensed sport physiotherapist with at least 2 years of experiences in managing ACL injuries? *

   *Mark only one oval.*

   ◯ Yes

   ◯ No

2. Your preferred contact method (E-mail or Phone Number): *

   ____________________

3. Your First and Last name (This is just for us to contact you regarding the interview) *

   ____________________

4. Age (in years): *

   ____________________

5. Sex *

   *Mark only one oval.*

   ◯ Female

   ◯ Male

6. Years of clinical experience you have? *

*Mark only one oval.*

- 0-5 years
- 6-10 years
- 11-15 years
- 16+ years

7. Role of current employment (e.g., researcher, clinician): *

*Mark only one oval.*

- Clinician only
- Researcher only
- Both clinician and researcher
- Other: ______________________

8. Highest qualification you compeleted: *

*Mark only one oval.*

- Bachelor’s Degree
- Master’s Degree
- PhD
- Fellowship/Board Certification
- Other: ______________________

9. Your primarily working sector/setting? *

*Check all that apply.*

- [ ] Governmental (Public hospital/Ministry of Health)
- [ ] Semi-governmental sector (e.g., KFMC, NGHA, KFSHRS, KSMC, SFHM)
- [ ] Academic institution (universities)
- [ ] Private clinics/centers
- [ ] Sport Clubs / Elite Athletes
- [ ] Other: ____________________

10. On average, how many ACL cases (with no surgery ) you dealt with last month? *

*Mark only one oval.*

- ( ) None
- ( ) 1-5 cases
- ( ) 6-10 cases
- ( ) More than 10 cases
- ( ) Other: ____________________

11. On average, how many post-ACL reconstruction cases you dealt with last month? *

*Mark only one oval.*

- ( ) None
- ( ) 1-5 cases
- ( ) 6-10 cases
- ( ) More than 10
- ( ) Other: ____________________

This content is neither created nor endorsed by Google.
